# Supplementary material for: Phylogeny of caucasian rock lizards (Darevskia) and other true lizards based on mitogenome analysis: Optimisation of the algorithms and gene selection
Source: PLoS One. 2020 Jun 8;15(6):e0233680. doi: 10.1371/journal.pone.0233680 (PMC7279592; doi:10.1371/journal.pone.0233680)

Fig. S1 Substitution saturation plot for individual genes of mitochondrial genome.

Nucleotide transitions and transversions versus divergence. The vertical axes is for the observed proportion of transitions (s) and transversions (v), respectively. Gene name is indicated on the each graf, genetic distance was applied according table 1.


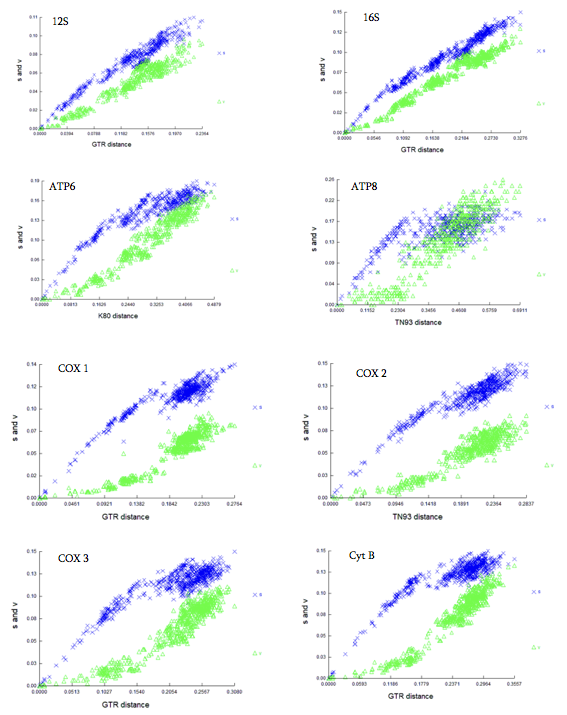


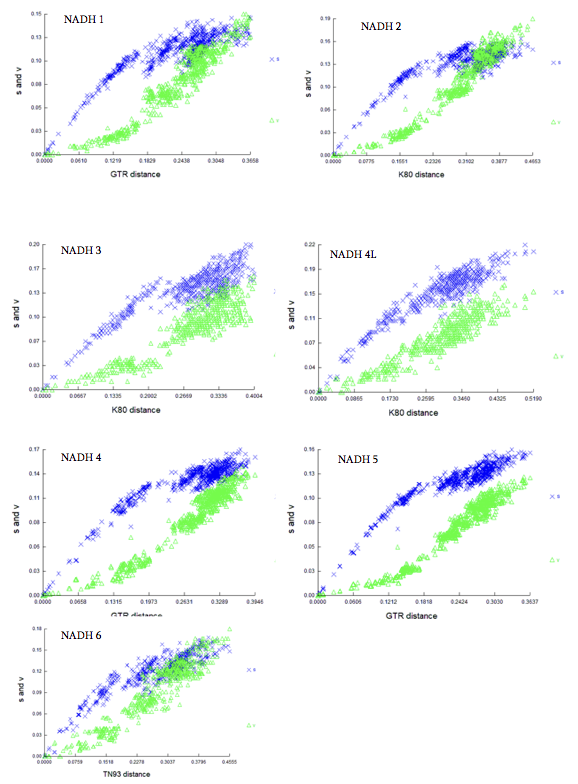

Supplement: S1 Fig — Nucleotide transitions and transversions versus divergence. The vertical axes is for the observed proportion of transitions (s) and transversions (v), respectively. Gene name is indicated on the each graf, genetic distance was applied according Table 1. (DOCX) [file pone.0233680.s001.docx]
