# Supplementary material for: Phylogeny of caucasian rock lizards (Darevskia) and other true lizards based on mitogenome analysis: Optimisation of the algorithms and gene selection
Source: PLoS One. 2020 Jun 8;15(6):e0233680. doi: 10.1371/journal.pone.0233680 (PMC7279592; doi:10.1371/journal.pone.0233680)
Supplement: Attachment — Submitted filename: Murtskhvaladze_responce to revieweres.docx [file pone.0233680.s002.docx]

*Dear Editor,*

*Thank you for evaluating our manuscript on the phylogeny of Darevskia. We learned in details the comments of both reviewers and re-wrote the manuscript respectively; in particular, in the presentation of the results and the discussion we concentrated on the genus Darevskia rather than on the all Lacertini, although maintained a broad selection of true lizards as the outgroups; only short statements were maintained on the outgroups, comparing with the findings of the previous authors, specifically basal position of Takydromus within Lacertini and sister status for Podarcis and Zootoka. We also removed the discussion on the potential factors of divergence among the Lacertini genera. The title of the paper has been modified.*

Dear Dr Murtskhvaladze,

Thank you for submitting your manuscript to PLOS ONE. After careful consideration, we feel that it has merit but does not fully meet PLOS ONE’s publication criteria as it currently stands. Therefore, we invite you to submit a revised version of the manuscript that addresses the points raised during the review process.

- *thank you. We substantially re-analysed and re-writed the manuscript according to the editor’s and the reviewer’s comments.*

Reviewer 1 judged your paper 'major revision needed' while reviewer 2 wanted to reject it completely. I follow here reviewer 1 in my decision. I do not think like reviewer 1 that phylogenies based on mitochondrial data alone have no value. But you should clearly discuss their limits. I also think that your data are worth to be publishes. But they are not complete enough for a good phylogeny of all Lacertini. Add more taxa or restrict your conclusions to those groups of which you have enough samples.

- *In the revised version of the manuscript, we restrict ourselves to the phylogeny of Darevskia and included only few critical points about the outgroups, not pretending on the reconstruction of phylogeny of true lizards.*

Please look carefully at all the points raised by both reviewers and re-write your manuscript completely. Discussion and conclusions should then be self-critical and show which conclusions are supported by your data and which not. I am looking forward to receive a new manuscript.

- *The MS is rewritten almost completely following the reviewers comments, and some comments are discussed below.*

5. Review Comments to the Author

Please use the space provided to explain your answers to the questions above. You may also include additional comments for the author, including concerns about dual publication, research ethics, or publication ethics. (Please upload your review as an attachment if it exceeds 20,000 characters)

Reviewer #1: The manuscript entitled PHYLOGENY OF CAUCASIAN ROCK LIZARDS (DAREVSKIA) AND OTHER TRUE LIZARDS BASED ON MITOGENOME ANALYSIS: RAPID RADIATION AND ALGORITHM DEPENDENT POSITION OF BASAL NODES is nice contribution presenting whole mitogenome based phylogeny of Darevskia and related genera of true lizards. Authors have sequenced and assembled 16 new mitochondrial genomes of Darevskia species, thus making possible to build phylogeny of Lacertini genera and resolve relationships inside Darevskia genus. The major finding in addition to a sequenced new mitochondrial genomes are detailed and well-grounded workflow of analysis of mitochondrial sequences including approach to minimize influence of substitution saturation. Unfortunately, these real advantages are not properly covered in the discussion and conclusions sections and instead too much space is devoted to discussion of topics which deserves to be a subject of separate research and instead has limited support from this particular study results, therefore the manuscript needs a major revision.

-*the authors tried to shift the emphasis in the discussion substantially, in particular they reduce the parts not directly linked with the findings.*

Authors did a good job comparing different approaches to whole mitogenome-based phylogeny reconstruction. One of the major findings is that saturation is the main obstacle in reconstruction of robust phylogeny and this could be overcome by careful selection of genes and outgroup or running analyses without outgroup at all. Unfortunately, this have not been reflected in the abstract.

-*we tried to discuss this in more detail in the revision, and shift the focus away from the discussion about geological past etc. (only retained in part that is directly related to the genus Darevskia).*

In addition, convergence of topologies obtained with different methods could be a good measure of robustness of phylogenetic reconstruction – authors should say it directly.

-*done*

Finally, I don’t see clear evidence of rapid radiation, just mitochodrial genome comes close to the limit of unambiguously reconstructing of ancient splits.

-*agree; the discussion on the potential rapid radiation removed as unnecessary.*

Introduction could be strengthen by adding information about possible flaws of mtDNA in phylogenetic reconstruction, namely only maternal inheritance, capture or lost by lineages via introgression, not-neutrality, functional ties with nuclear genes, possible presence of pseudogenes etc., and how at least some of these difficulties can be targeted when using complete mitochondrial genomes. Please, check these papers
Podnar, M., Haring, E., Pinsker, W., Mayer., W. 2007. Unusual origin of a nuclear pseudogene in the Italian wall lizard: Intergenomic and interspecific transfer of a large section of the mitochondrial genome in the genus Podarcis (Lacertidae). J. Mol. Evol. 64, 308-320.
Godinho, R., Crespo, E.G., Ferrand, M., 2008. The limits of mtDNA phylogeography: complex patterns of population history in a highly structured Iberian lizard are only revealed by the use of nuclear markers. Molecular Ecology. 17, 4670–4683.
<https://www.pnas.org/content/99/25/16122?cited-by=yesl99%2F25%2F16122&utm_source=TrendMD&utm_medium=cpc&utm_campaign=Proc_Natl_Acad_Sci_U_S_A_TrendMD_0>
<https://www.biorxiv.org/content/10.1101/588756v2.abstract>

-*these issues are addressed in the revised introduction*

I think the manuscript should be substantially revised to be more concise, and keep track on the main ideas whose are supported by results. This includes changing title of the paper to more appropriate (rapid radiation is not the only possible reason for absence of strong support of basal nodes; perhaps loosing of phylogenetic signal and non-monotonous rates of molecular evolution among species could also contribute).

- *agreed, the title is changed, and the relevant issues addressed in the revision*

Other remarks to the text (unfortunately, lines numbering in PDF was missing):

-*lines numbering inserted in the revision*

“inter-generic phylogenies.” - there is extra return in Abstract

“but BI and ML topologies support basal position of D. parvula from the western Lesser Caucasus.” - did you mean BI and MP?

-*of course, thank you for the correction.*

“All topologies shifted the phylogenetic position” - did you mean altered, shuffled?

- *altered; corrected*

In Methods “The sum of the conflicting, coinciding, and unsupported nodes was considered a measure of similarity between the compared trees” - haven't you consider plotting some of these numbers against saturation indicator to show it’s influence?

*-thank you for this suggestion! In the revision, the correlation between the conflicting node number and saturation index is discussed.*

The problem of reconstruction of phylogeny using saturated mitochondrial genes seems not new – see for example <https://www.ncbi.nlm.nih.gov/pubmed/11975355> <https://www.ncbi.nlm.nih.gov/pubmed/29669515> and it should be discussed

- *we addressed the issue in the revision*

Fig. 2 legend “Posterior probabilities left of the nodes” does not seem grammatically correct

-*corrected*

“However, genus-level topologies were still different (results not shown).” - you have Supplementary files where you can show these results.

-*we don’t discuss genus-level topologies in the revision, with the exception of two important patterns: basal Takydromus and sister status of Zootoka and Podarcis.*

There is subsection “Differences from earlier findings” in Discussion. I think it should be shortened – obviously lots of reconstructions had been made, there is no need to discuss differences in such detailed way.

- *the discussion is reduced substantially, according to this recommendation. Besides, we minimized the statements on the Lacertini phylogeny, considering the recommendation of the 2^nd^ referee and the editor, although retained few points that we think are important and well-supported.*

I am surprised not to see molecular dating reconstruction. Authors definitely have very good dataset for that, moreover, they are studying one taxonomic group, which is very much the group of their expertise – why not? It would add value to the publication and justify discussion of biogeographic scenario if it will persist in the final version.

-*we prefer to be careful about the dating, since there is very broad range of calibrating molecular clock for lacertids by different authors. However, following the suggestion of the referee, we used two alternative datings, more and less conservative, referring on two important geologicasl events shaping the region: middle Miocene climate transition and Messinian salinity crisis. The respective datings are discussed and illustrated.*

“In the Western Lesser Caucasus area, D. parvula coexists with the representatives of the
next basal clade of Darevskia: the clade ‘D. rudis’ (in sense of (1). Consequently, this area is the
most likely ancestral for all Darevskia. In the original time of divergence, this was the north-east-
ern part of the Anatolian landmass, and the separation could have happened between the water-
sheds separating the upper reaches of the Kura (Mtkvari), Chorokhi (Coruh), and Euphrates Rivers,
due to the growth of xeric and less suitable areas between the lizard habitats.” - Authors may use more formal approach to the ancestral range calculation. Currently, this discussion looks speculative, especially the part with discussion of climate of watersheds. 20 Mya the area could look different due to massive transgressions-regression cycles there – see Popov, Rögl, Rozanov_2004_Lithological-Paleogeographic Maps of Paratethys Cour. Forsch.-Inst. Senckenberg, 2004. — 250 p. Authors definitely must to do additional analyses inferring split time, ancestral areas, ecological features of species of Darevskia genus (ground dwelling versus rock dwelling) and rewrite correspondent sections of manuscript or omit these topics. Actually I would suggest to concentrate here only on molecular dating, since the last two issues would benefit from additional taxa, which are not included in this study.

-*we followed this suggestion working on the revision.*

Conclusions must be rewritten according to the new version of manuscript. As an example only, currently “The first is that constructing phylogenies based on the analysis of high volumes of various genetic data is not the best approach for reconstructing species-level phylogenies.” is highly ambiguous;

-*Conclusions section re-written*

“Phylogenetic studies devoted to a specific taxonomic group, such as genera or subfamily, are more trustful for resolving species-level taxonomies than meta-analyses comprising large number of taxa with different evolutionary distances from each other.” is trivial and too general and so on.

-*agree, changed*

The last part of last sentence “however some elements of topology are unlikely to be finally resolved even if the complete mitochondrial genome is studied, and, if high-throughput sequencing is not available, it is more useful to combine sequences of mitochondrial and nuclear genes, as (66) recommend.” is merely speculative and is not conclusion of this research.

-*removed*

Reviewer #2: In the present manuscript the authors sequence 16 mitochondrial genomes of the lacertid genus Darevskia and use them to reach conclusions of the phylogenetic relationships within this genus and, together with GenBank sequences, to infer the phylogenetic relationships within Lacertini. Although sequencing 16 mitochondrial genomes is a good effort it is certainly not the way forward to solve evolutionary relationships in the XXI century. The mtDNA is very long an can provide quite a lot of information but the main drawback is that it represents a single locus. In fact, mtDNA is a very particular locus with exclusively maternal inheritance and with a high evolutionary rate. Aspects that have proven very good for resolving many evolutionary relationships but that have also been shown to recover wrong phylogenetic relationships in many occasions. For this reason, in the last 10 years it has been very usual to apply a multilocus approach to phylogenetic inference, first with a few loci (2-3) but latter on with more (5-8) and more recently with hundreds or even thousands of loci. Of course, missing data is still an issue but multilocus phylogenies are certainly the way forward to obtaining the correct evolutionary relationships of taxa. Especially on these groups in which the phylogenetic relstionships are contentious. NGS techniques have opened new doors to dig further into the phylogenetic relationships of difficult groups (including Lacertini) using many independent loci. To suddenly apply these NGS techniques to obtain long stretches of a highly variable and maternally inherited single locus is like going back to the beginning of phylogenetic inference. In fact, one of the conclusions of the present manuscript is that just to genes, 16S and cytb, give the same (or very similar) results than the whole mitogenomes. Phylogenies of 16S and cytb have been around for more than 20 years and luckily technical and analytical advances occurred in the last 10 years have allowed a leap forward towards multilocus phylogenies, a much better approach than single locus phylogenies like the one presented in the present study.

- *We know that it is possible to sequence nuclear genes and also use genomic data. However, (1) multilocus approach has its own shortages for reconstruction of low-level phylogenies, where gene introgression commonly takes place, or incomplete lineage sorting may be the case. In fact, mitochondrial DNA phylogeny may not completely coincide with species-level phylogeny but its interpretation is straightforward – this is phylogeny of maternal lineages. This is indicated in the text. (2) as mentioned in the paper, there are hundreds of DNA laboratories worldwide that are and will be during quite long time rely on PCR based methods and individual gene sequences. If answers on some specific questions can be found with less equipment and cheaper techniques, we should not reject this opportunity.*

In the manuscript the authors use a largely incomplete phylogeny of Lacertidae to discuss the phylogenetic relationships within Lacertini. The results presented are completely uninteresting and definitively not worth publishing. The authors should delete this whole section from the paper. There have been recent studies using multilocus data that have not been included in the manuscript (Mendes et al. 2016 Molecular Phylogenetics and Evolution 100 (2016) 254–267) and this makes the whole discussion comparing to papers by Fu et al. and Harris et al from 1997 and 1998 and other older papers irrelevant and uninteresting. The authors are missing many Lacertini genera and therefore they should not use their incomplete dataset to discuss the phylogenetic relationships within this very relevant group. Doing that is a superfluous and confusing exercise that is, again, not worth publishing. After Mendes et al. 2016 the phylogeny of Lacertini is more or less fixed until new studies using thousands of loci obtained using NGS will be published in 2020. Datasets should be developed to answer specific questions and this partial mitogenomic dataset is definitively not suitable to address the phylogeny of Lacertini.

-*the paper of Mendes et al. is cited in the revision. The discussion on the inter-generic relationships of Lacertini is strongly reduced.*

Across the whole manuscript the authors use the word “basal” incorrectly. I urge the authors to read this paper very carefully and correct the way they discuss phylogenies.
<https://www.csun.edu/~dgray/Evol322/OmlandTreeThinking.pdf>

-*The authors are familiar with the basics of cladistics philosophy, and they did not use the word “basal” in sense of “ancestral” which seems to be the concern of this reviewer--Basal is used in sense of the branch or clade in the phylogeny most distant (and earliest separated) from the rest of the evolutionary lineages. We do not agree with the reviewer that this term is used incorrectly throughout the manuscript.*

The exercise within Darevskia is interesting but again it is restricted to half of the described species. In fact, they only include species within Georgia.

- *The species set included in the analysis represents all clades and major groups of Darevskia, except group D. defillippi. There are species of the genus found far south, west, and east from the sampling area, however, they are either found in Georgia as well, or their closest relatives are found there. The only group of species not found in Georgia is D. chlorogaster, and the sample of this species collected from SE Azerbaijan is included. Because all of the major clades are well represented, and because relationships of the more terminal taxa are not at issue, we feel that the species presented in this analysis provide an optimal assortment of Darevskia taxa, commensurate with our limited resources and time.*

Minor comments:
P12: Neither in the Fig. legend or in the figure itself the authors indicate Georgia. They should indicate where is Georgia.

-*corrected*

P14: The D. parvula is most probably D. adjarica (previously D. parvula adjarica). See <https://biotaxa.org/Zootaxa/article/view/zootaxa.4472.1.3>

-*according to* *the Reptile Database (*<http://www.reptile-database.org/>*) it is still D. p. adjarica, we rely on this nomenclature.*

P14: D. clarcorum is wrongly written in several places in the text (OK in the map). It is d. clarkorum, with “k”.

-*corrected*

Pag 14: “E. multcocellata” is E. multiocellata

-*corrected*

Pag 17: in several places they use “polytiomies”, correct to “polytomies”

-*corrected*

Pag 21: Legend for Fig. 4 “genes” is repeated twice

Within Darevskia, “parvula” (or better adjarica; see Arribas et al. 2018) is sister taxon to the remaining Darevskia included in the study. Despite they present it as a new finding, it had been already published by Fu et al. (1997) and mentioned by Arribas et al (2018).
Please, check this paragraph from Arribas et al. (2018):
“Grechko et al. (1997) found that D. parvula was sister taxon to all the other Darevskia when studied using “DNA Taxon prints” (Restriction endonuclease analysis of Highly Repetitive DNA). Ciobanu et al. (2002, 2003) and Grechko et al. (2006) also studied Satellite DNA in Darevskia and found a relatively isolated position of D. parvula in which only one type of Satellite DNA predominate (CLsatIII), and as inhabitants of one of the glacial refuges in the extremes of the Caucasus (as D. clarkorum/dryada) in the west and D. chlorogaster in the southeast, they argue that possibly they resemble the ancestors of the group. The presence of these same microsatellites in the “mixta-group” (clarkorum, caucasica, daghestanica, dryada) casts some doubts on the basal association of D. parvula with the rudis group as stated by means of mtDNA (see above). For Grechko et al. (2006), D. parvula would be outside these two clades, but hybridized in an early instance of its diversification with the ancestors of both clades.”

- *Thank you for this comment. Fu et al. (1997) suggested basal position for D. praticola, and not D. parvula. Grechko et al. indeed hypothesized this based on the microsatellite and restriction endonuclease analysis, and we mention this in the revision.*

-At the end of page 17 they comment: “We did not include D. alpina in our analysis, however, this species is matrilineally very close to D. saxicola, and may be of hybrid origin”. What is possibly of hybrid origin is not the species but the individual sewuenced by Murphy et al. To extrapolate it to the whole species is a bit excessive.

- *the statement is re-worded.*

Page 27 etc. As mentioned in the general comments above, the manuscript is not suitable for addressing the phylogenetic relationships and/or the biogeography of lacertini. It is missing many taxa, it uses a single locus and therefore it is wrong and confusing. I would suggest the authors to refrain from publishing these results on the phylogeny and biogeography of Lacertini

-*as said above, the respective sections are removed or greatly reduced*
