# Supplementary material for: Phylogeny of caucasian rock lizards (Darevskia) and other true lizards based on mitogenome analysis: Optimisation of the algorithms and gene selection
Source: PLoS One. 2020 Jun 8;15(6):e0233680. doi: 10.1371/journal.pone.0233680 (PMC7279592; doi:10.1371/journal.pone.0233680)
Supplement: Attachment — Submitted filename: replies to comments march 2020.docx [file pone.0233680.s004.docx]

Additional Editor Comments (if provided):

Please obey the requests by reviewer 2 and add some explanation of the tree.

- *the request of the reviewer 2 considering the terminology is considered, and the time-tree is additionally explained in the methods subsection 2.4.*


Reviewer #1: Line 196 “were:Podarcis” - space missing

- *corrected*

L. 203 “E. multcocellata” – again!

- *Of course, multiocellata. Corrected.*

Line 214 “);,” - delete semicolon

- *corrected*

Line 269 “D. Clarkorum” - unnecessary capitalization

- *corrected*

Line 474 “odd Castoe” sounds odd

- *of course, Todd Castoe. Corrected.*

Sorry to say, but what I am still missing is the description of methods and results of time-calibrated tree. The Figure 6 pops up from nowhere first time already in Discussion section. Please, add few lines how this analysis was done and what is showed in terms of topology of tree, convergence of parameters, dates etc. I believe, that this is my last request.

- *For inferring time-calibrated tree we used the same BEAST runs as for reconstructing full-mitogenome-based BI phylogeny, using node calibration suggested by Garcia-Paris et al. We added few lines to the Methods subsection 2.4 to explain this.*

Reviewer #2: As mention in my previous reviews, I do not like the present manuscript. I think that by being mtDNA only it is more misleading than helpful; even if what is use are mtDNA genomes. Said that, the authors have done an effort to include most of the comments by the referees. They still are wrong with the use of the term "basal" but as I said in my previous reviews with this and many other errors that they had: it is not my paper and therefore it is up to them to fix it. Please have a look to the following paper:
<https://onlinelibrary.wiley.com/doi/full/10.1111/j.0307-6970.2004.00262.x>

- *The authors discussed the use of the term “basal” and decided to follow the recommendation of the reviewer. In the revised version, we are speaking about “basal branchings” rather than basal taxa.*
